# Burnett’s Disinfecting Fluid

**Published:** 1847-10

**Authors:** 


*Burnett's Disinfecting Fluid.*—The chloride of zinc in solution, it appears from a parliamentary document which has just been issued, has been employed extensively as a disinfectant in dissecting-rooms, the wards of hospitals, and in the royal navy, and, according to the reports which we have seen, has been eminently successful in effecting the objects for which it is designed. The medical officers at Haslar Hospital state that it has been used in that hospital in the close stools of patients affected with dysentery, in the water-closets and cesspools, and also in the wards, when the air was tainted by purulent expectoration or discharge from sores, with the effect of immediately removing the disagreeable odours. It has also been used in surgery with good effect, in removing the smell of putrefying animal substances, and the odour of dead bodies under inspection: when employed as a dressing to ulcers, it removes the disagreeable smell of purulent matter, and, in the proportion of one part of the clear solution to eighteen of water, it preserves subjects of natural history from putrefaction, and in a fit state of anatomical inspection, after more than a year has elapsed. A similar testimony in favour of the solution of the chloride, is borne by the assistant surgeon of the Marine Hospital at Woolwich, who adds, "the great advantage which the chloride of zinc possesses over other agents employed for a like purpose, is, that it removes the disagreeable effluvium, without leaving one little less offensive in its room, and may therefore be made use of wherever this effect is required—in private as well as public buildings, in the sick bed chamber no less than in the crowded ward. The method adopted at this hospital is to supply each of the wards with a bottle of the diluted solution, which the nurses have directions to use whenever occasion may require, besides sprinkling it over the floors before the morning and evening visits are made.

Its utility in the dissecting-room is confirmed by the statements made by Mr. Bowman, Dr. Sharpey, Mr. Partridge, Dr. Murray, and Dr. V. Pettigrew, who concur in asserting, that in a proper degree of dilution its success is complete, and that it appears to preserve the colour and texture of the parts very admirably. It has, further, the very important advantage of not acting on the steel instruments employed, being in this respect equal to alcohol. Dr. Methven especially mentions an instance in which the solution corrected advancing putrescence, and enabled him to dissect during July. He believes, further, it will be the means of saving many valuable lives, which are annually lost by wounds received in the course of dissection, as, while dissecting this putrid body, he cut himself several times, and once received a punctured wound, without any bad consequences arising. Mr. M'Bain, of the "Mastiff," adds his testimony "to the rapid and perfect effects of the chloride of zinc solution upon animal matter in a state of putrefaction. Having frequently opportunities of dissecting or examining large fish, &c., cast on shore, whilst undergoing decomposition, the task has been occasionally any thing but agreeable, for want of a convenient power to destroy the putrefactive process. The chloride in these cases acts like magic; and as a great practical agent over one of the most import conditions of animal and vegetable matter—viz: putrefaction, it stands unrivalled." Its influence on board ship, in annihilating the offensive smell of bilge-water, and in sweetening between decks, is shown by the united evidence of captains, surgeons, and masters in the royal navy. Among other vessels, it was used on board the "Victoria and Albert" royal yacht, to remove a more than ordinary stench of bilge-water, and other offensive odours, with the most complete success. The surgeon states that she has remained comparatively sweet ever since, and when a bilge-water smell is occasionally perceptible, a slight application of the fluid removes it. The solution has also been used for very disgusting privies, &c., effluvia from which, it quickly neutralizes.

Mr. Henderson, the surgeon to the dock-yard at Portsmouth, has employed the fluid in a severe case of open cancer, the fœtor from which was intolerable to the patient and attendants: this it destroyed so long as the dressings were kept moist therewith. Professor Quain has used it, he says, in the treatment of sloughing tumours with beneficial result, and he has no doubt it will supplant the chloride of lime and soda altogether in the removal of fœtid odour. Mr. Gibson, surgeon of the "Eurydice," employed it in a case of angry ulcer, in the proportion of one part to four of water. An eschar was the result, the separation of which left the ulcer in a healthy condition.

Several naval and other medical men have employed it as a disinfectant in hospitals, and on board ship, the general results being a marked diminution in the rate of mortality. Dr. Lindsay, Dr. Cronin, and Dr. Connor, of Cork, all bear testimony to its beneficial effects. Mr. Verling, surgeon of the "Vengeance," thus speaks:—

"Having used the chloride of zinc rather extensively on board Her Majesty's ship 'Vengeance,' whilst employed in the conveyance of troops, I think proper to report to you the result thereof. We carried the first battalion of the forty-second regiment, consisting of about 700 men, women, and children, from Malta to Bermuda. Measles had prevailed epidemically in the regiment previously to their embarkation, but we received none on board labouring under the disease; yet after being ten days at sea, several cases occurred simultaneously among the soldiers, and on the 1st of April, having been then a month at sea, the disease appeared among our own people, ten cases occurring on that day, and from that day to the fifteenth of the month, when we arrived at Bermuda, fresh cases were almost of daily occurrence, either among our own people or the troops. On getting rid of the troops, which we did at Bermuda, my attention was of course specially directed to every means whereby the contagion could be destroyed. Cleanliness and ventilation were duly attended to, and every part of the ship where the sick had been, after being cleaned and aired, was sponged well over with the solution of chloride of zinc several times. Than the result, nothing could be better; the disease totally ceased, no fresh case occurring after. On our passage from Halifax, with the sixtieth regiment on board, the weather was so bad, and the ship working so much, that it was quite impossible to open any of the lower-deck ports, on which deck the whole of the people lived, troops as well as our own people, for eight days; the air throughout the deck was exceedingly vitiated with every mixture of noxious smell, but the free use of the chloride of zinc tended, in a most surprising manner, to do away with the bad smell; so much so, that the surgeon of the regiment came to me to get some to use in the part of the ship where the ladies of the officers were.

The effect of the chloride of zinc is most obvious in correcting all bad and offensive effluvia; and from the sudden and surprising manner in which the measles disappeared after its use, it is not, I think, too much to say, that it must have been very instrumental in decomposing the miasma, or state of atmosphere in the ship, which tended to the generation of the disease."

From all these statements, then, it is clear that the solution of the chloride of zinc is a powerful agent in neutralizing noxious gases, and in arresting the progress of decomposition. Sir W. Burnett has therefore rendered, by its discovery, a great benefit to suffering humanity. On board ship, its influence in removing the offensive odours from bilge-water can hardly be too highly estimated, while its action in sweetening the wards of hospitals, and destroying noxious and infectious effluvia, seems to be equally evident.—*London Lancet.*

